# Environmental monitoring — a flow–injection approach

**DOI:** 10.1155/S1463924694000180

**Published:** 1994

**Authors:** Paul J. Worsfold

**Affiliations:** Department of Environmental Sciences Universily of Plymouth Plymouth PL4 8AA UK


Journal of Automatic Chemistry, Vol. 16, No. 5 (September–October 1994), pp. 153–154

### Environmental monitoring—a flow-injection approach*

**Paul J. Worsfold**

*Department of Environmental Sciences, University of Plymouth, Plymouth PL4 8AA, UK*

### Water quality

Water quality is a subject of increasing public interest and awareness. With regard to chemical parameters in particular it is also an area that has been the subject of increasingly stringent national and international legislation based on EC directives, for example, for the discharge of dangerous substances into the aquatic environment (EC/76/464) and water quality for human consumption (EC/80/778).

This has necessitated the development of reliable analytical methodologies that meet specified requirements for accuracy, precision and limit of detection, and, if possible, can be automated for high sample throughputs. The conventional approach is to combine periodic manual sampling with automated laboratory analysis. The most labour-intensive and rate-determining step in the procedure is sample collection, which can also introduce problems due to contamination and/or analyte loss during collection, pretreatment and storage.

### Field monitoring

It is clear that there is a need for *in situ* analytical techniques for monitoring water quality. Not only would such systems overcome the problems stated above, they would also provide a more immediate response (useful in the case of transient pollution incidents) and a more complete concentration versus time profile (useful for archiving and management purposes). The latter point is a particularly important one for fundamental environmental studies when other aspects of the local environment are also monitored pseudo-continuously *in situ*, for instance, flow rate, temperature and salinity.

It is, however, very difficult to adapt laboratory instrumentation for reliable field use. In addition to the analytical requirements specified above, a field monitor must also consider the following:.

(1) Reagent stability.

(2) Hardware and software flexibility.

(3) Internal diagnostics.

(4) Stay-clean properties.

(5) Modular construction.

(6) Long-term unattended operation.

(7) Periodic recalibration.

(8) Easy on-site maintenance.

### Analytical approaches

Ion-selective electrodes represent an analytical technology suited to *in situ* monitoring due to cost and size advantages but they are only available for a limited number of determinands, for example, nitrate and ammonia, and are prone to fouling of the sensor surface and base-line drift. Biological monitors, i.e. the use of living organisms such as fish and daphnia, present an interesting possibility for the future and function on the basis of increased movement or ventilatory frequency in the presence of pollutants. They are, however, not sufficiently selective to provide information on individual chemical species.

Spectrophotometric detection is potentially very attractive for field use because there are well-documented methods for most organic and inorganic species that utilize a wide range of selective and sensitive reagents. Conventional spectrophotometric detectors are too expensive, complex and heavy for remote deployment, but solid state detectors (SSDs) based on light-emitting diodes (LEDs) as sources and a photodiode as the detector provide a rugged, compact and low-cost alternative.

Flow injection (FI) analysis is an ideal means of presenting sample to the SSD (and undertaking on-line sample treatment if necessary) and offers a number of desirable features:

(1) Rapid response.

(2) Economical use of reagents.

(3) Flexible sample pretreatment.

(4) Flexible manifold design.

(5) Low purchase and operational costs.

A block diagram of an FI monitor suitable for remote deployment is shown in figure 1. A single board computer is used for control and data acquisition and a standard is available for regular recalibration via a switching valve.

Sample presentation to the monitor is a crucial aspect of the system and can range from a simple constant head device for treated waters to a sophisticated combination of filtration steps for high suspended solids waters. Biofouling is another important factor that must be considered, particularly for long-term, i.e. greater than seven days, unattended operation. The importance of regular (short) maintenance visits cannot be overemphasized.

* *Inaugural lecture: University of Plymouth.*

0142-0453/94 $10.00 © 1994 Taylor & Francis Ltd.

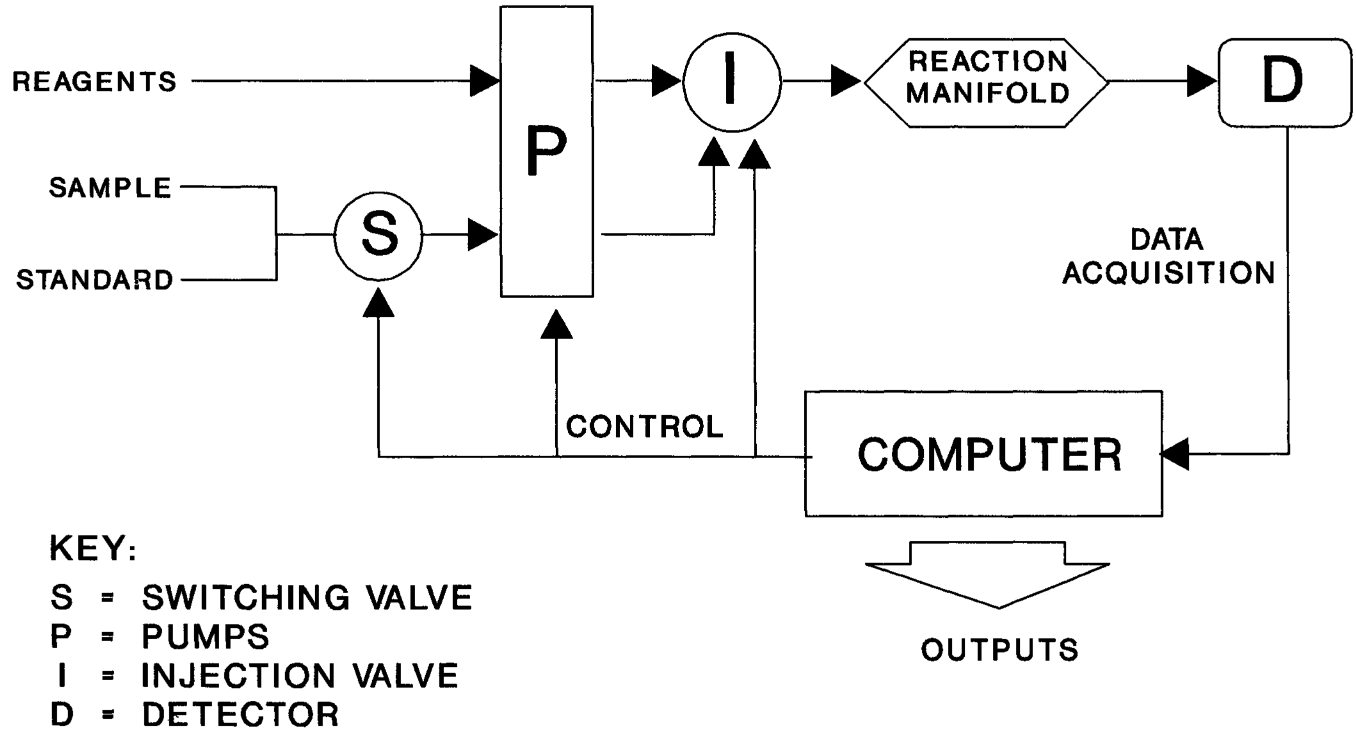

*Figure 1. Automated FIA monitor.*

Data transfer from the monitor must also be considered, but there is a range of options available including immediate transmission via telemetry links to a central facility and storage of data for periodic collection via a data logger. A local readout is also useful for diagnostic purposes.

### Applications

A number of applications of FI-based instrumentation with SSD have been reported for *in situ* monitoring. These include the determination of nitrate [1–3], phosphate [4] and ammonia [5] in river water and the determination of aluminium [6] and iron [7] in treated waters. There is also considerable potential for the remote deployment of similar instrumentation in harsh industrial process environments [8].

### Conclusions

FI with spectrophotometric detection is well suited to remote deployment in environmental locations for the pseudo-continuous and selective monitoring of chemical species in natural and treated waters.

### Bibliography

1. Clinch, J. R., Worsfold, P. J. and Casey, H., *Analytica Chimica Acta*, **200** (1987), 523.
2. Clinch, J. R., Worsfold, P. J., Casey, H. and Smith, S. H., *Analytical Proceedings*, **25** (1988), 71.
3. Casey, H., Clarke, R. T., Smith, S. M., Clinch, J. R. and Worsfold, P. J., *Analytica Chimica Acta*, **227** (1989), 379.
4. Worsfold, P. J., Clinch, J. R. and Casey, H., *Analytica Chimica Acta*, **197** (1987), 43.
5. Clinch, J. R., Worsfold, P. J. and Sweeting, F. W., *Analytica Chimica Acta*, **214** (1988), 401.
6. Benson, R. L., Worsfold, P. J. and Sweeting, F. W., *Analytica Chimica Acta*, **238** (1990), 177.
7. Benson, R. L. and Worsfold, P. J., *Sci. Tot. Env.*, **135** (1993) 17.
8. MacLaurin, P., Worsfold, P. J., Townshend, A., Barnett, N. W. and Crane, M., *Analyst*, **116** (1991), 701.

